# A Radiologist Visits Africa

**Published:** 1954-07

**Authors:** J. H. Middlemiss

**Affiliations:** Consultant Radiologist, United Bristol Hospitals


### A RADIOLOGIST VISITS AFRICA

BY

J. H. MIDDLEMISS, M.D., F.F.R., D.M.R.D.
*Consultant Radiologist, United Bristol Hospitals*

In February 1953 I set out on a three-months trip for the Colonial Office. I visited Sierra Leone, the Gold Coast, Nigeria, Northern Rhodesia, Tanganyika, Zanzibar, Kenya and Uganda; and in addition to these territories under the administration of the Colonial Office, I also visited the Union of South Africa and Southern Rhodesia. The object of the trip was to meet anybody who was practising X-ray work in any form, to discuss their problems with them, and if possible to help them. Such help ranged from showing a medical officer in a bush station how to use his X-ray apparatus to advising a Government what apparatus to buy, and from trying to arrange maintenance services on a zonal basis for Africa to interpreting the films of abstruse diseases. It was useless to think of X-ray work for say, Nigeria or Tanganyika in the same terms and on the same scale as in this country. It was necessary first to see radiology in relation to the type of work carried out in the hospitals and it was necessary to see hospital practice against the background of community life, social conditions, tribal customs, standard of education and so forth. As a result I spent much time in bush villages watching meals being prepared in mud-huts, in shanty towns looking at living conditions, with sanitary inspectors seeing conservancy schemes or malaria control schemes, and with Ministers of Health discussing their plans. And in order to get a true impression of the pattern of disease I spent as much time seeing patients in native dispensaries and hospital " casual " or out-patient departments as I spent going round hospital wards.

The pattern of disease that came within my sphere fell broadly into two categories—trauma and pulmonary tuberculosis. Pulmonary tuberculosis is widely disseminated particularly where life has become at all " urbanized ". At present the African appears to have little resistance to tuberculosis and a progressive primary infection with cavitation is a common response to infection. Often by the time it comes to light the disease has progressed far beyond a stage from which there is any reasonable chance of recovery. At present, the extent of the problem is not even known; a survey is being planned in the Gold Coast to determine how widespread pulmonary tuberculosis is in the territory; similar surveys are under way in various parts of Nigeria and also in Northern Rhodesia. It is hard for us in this country to realize what problems such a survey may produce; first of all there are difficulties in persuading tribal peoples to submit themselves to X-ray examination, and in getting them to co-operate in such a simple procedure as holding their breath; and there is the by-no-means small problem of carrying heavy " mobile " X-ray equipment where no roads or bridges but only dirt tracks and ferries exist. Previously no one radiologist had visited all these territories. But now the experience gained in one territory can be put to use in another, the teething troubles with new apparatus in one place can be avoided in another, and tribal difficulties in one area can be anticipated elsewhere, and so forth.

Trauma, the other major source of X-ray work is found in all territories. In many hospitals " lorry accident " is a diagnosis, and the most appalling examples of fractures are to be seen wherever there is motor traffic. Railways and docks, too, produce their share of fractures. In Sierra Leone and again in Zanzibar, I met what amounted to an occupational disease—fracture of the spine sustained by falling from palm-trees while collecting either palm-kernels or coconuts.

Apart from trauma and pulmonary tuberculosis, X-ray work varies from place to place depending on the tribal customs, local industries and the interests of the local doctors. If, for instance, the surgical specialist in a centre is interested in repairing vesico-vaginal fistulae he will find unlimited clinical material and he will call very little on the X-ray service. If, however, he is interested in orthopaedics, again he will have unlimited clinical material, and in addition he will make heavy demands on the X-ray service. If the local industry is banana growing as in Uganda, or clove production as in Zanzibar, or cocoa growing as in Ashanti province of the Gold Coast, there will be little in the way of industrial disease. But in the copper belt of Northern Rhodesia, where mining is extensive, silicosis is a very real problem. An elaborate Silicosis Bureau has been established at Kitwe with modern X-ray apparatus and a team of experts who work entirely on this problem. In Tanganyika there are gold-mines south of Lake Victoria; so far it is not known whether there is any silicosis there. But a scout survey of some 500 miners is being carried out along lines suggested by me and I shall interpret the films. From this it is hoped to learn whether a silicosis problem exists and whether or not any preventive measures are to be taken. Such a survey, of course, is not the simple procedure that it would be in this country; there is no available electricity supply so a small petrol-engine generator has to be carried; the Africans being examined are suspicious and not always co-operative; X-ray film fogs easily under these conditions and has to be treated carefully; there is no dark-room available and a mud-hut has to be adapted to the occasion; processing of exposed X-ray films should be carried out at a temperature of 65–68° F.—under these conditions it will have to be done at about 85° F.

The recruitment of technical staff from home or the training of Africans to use X-ray apparatus are both subjects which cannot be regarded as simple straightforward problems. I visited some hospitals for instance where the very busy medical officer was trying among his many duties to do the X-ray work. Often he had not been trained for the work nor could he always interpret the results; and usually he had insufficient time to devote to it anyway. The answer in such places would be temporarily to recruit a European radiographer to do the technical work. But a European technician would need a house to live in and this building would be low on the priority list, for in some instances there are not houses even for doctors (in one Mohammedan village I found the doctor living in an " African round-house "). It would take three years to train an African for the work provided a vacancy can be obtained for him in one of their few training schools. Few natives have the right educational standard. In Nigeria, for instance, in a population of 30,000,000 there are 30,000 people with School Certificate; they are the "cream " of the educated people and they are or will become the professional classes, so they are not prepared to remain technicians; those who have not reached School Certificate standard are usually far behind, or have had no schooling at all; they are unable to read or write, and sometimes they cannot even speak English. Obviously it is not possible to teach them the elementary anatomy or apparatus construction to cope with even the simplest aspects of this technical work.

The countries that I visited are developing rapidly and African consciousness is awakening everywhere. But in many ways Africans are still centuries behind us; and they have not yet the education, money or facilities to benefit from all the advances of modern medicine. We have a moral obligation to train these people, to guide them and to let them have the fruits of our experience. In the field of radiology, one of the most expensive in the whole of medical practice, they need much sound advice and guidance so that the plans are well laid and the growing facilities are used to the best advantage.

